# Hyperhomocysteinemia as a risk factor for cerebral venous thrombosis: a systematic review and meta-analysis of the case–control studies

**DOI:** 10.1097/MS9.0000000000004861

**Published:** 2026-04-01

**Authors:** Nawris Alhassan, Jagdish Lal, Susmin Karki

**Affiliations:** a Biomedical Sciences, Hope Clinic, Midvale, USA; b Physical and Occupational Rehabilitation, Burnaby Chronic Pain & Rehabilitation Clinic, Burnaby, Canada; c Institute of Medicine, Tribhuvan University Teaching Hospital, Kathmandu, Nepal

**Keywords:** cerebral venous thrombosis, hyperhomocysteinemia, meta-analysis, risk factor, systematic review

## Abstract

**Introduction:**

Cerebral venous thrombosis (CVT) is relatively rare but has severe thrombotic manifestations with a high mortality rate, the potential to cause disability, and the propensity to recur. There are limited data on the role of hyperhomocysteinemia as a risk factor for CVT. In this context, no large-scale meta-analysis has been conducted. Our meta-analysis aims to assess hyperhomocysteinemia as a risk factor for CVT using the data from case–control studies.

**Methods:**

We conducted a meta-analysis to investigate hyperhomocysteinemia as a risk factor among patients with CVT. A literature search was conducted using PubMed, Embase, Google Scholar, and Scopus databases until August 2025. A meta-analysis was performed using high-quality case–control studies that compared the homocysteine levels in patients with CVT and healthy controls. At least two authors extracted summary data. Between studies, heterogeneity was assessed using the *I*^2^ statistic. For each exposure–outcome pair, a random or fixed effect meta-analysis was conducted based on the degree of heterogeneity to pool the odds ratio (OR), prevalence of hyperhomocysteinemia, and the mean difference (MD) in homocysteine level with a 95% confidence interval (CI) from the individual studies using RevMan version 5.4.

**Results:**

This meta-analysis included six eligible studies. These studies involved 1355 participants (455 CVT cases and 900 controls). There were 123 men in the CVT group and 295 men in the control group. There was a statistically significant increased risk of CVT among patients with hyperhomocysteine levels (OR = 4.17, CI = 2.34–7.43, *P*-value < 0.00001, *I*^2^ = 70%). Furthermore, the homocysteine level was significantly higher in the CVT patients compared with the healthy controls (MD = 7.00, 95% CI = 3.21–10.79, *I*^2^ = 88%, *P*-value = 0.0003). The prevalence of hyperhomocysteine levels among CVT patients was higher compared with healthy controls (37%, 95% CI = 29–46%, *I*^2^ = 66%) versus (15%, 95% CI = 9–23%, *I*^2^ = 90.4%).

**Conclusions:**

In conclusion, this meta-analysis demonstrates a strong association between CVT and hyperhomocysteinemia, with a notably high prevalence among CVT patients. While these findings suggest that hyperhomocysteinemia may be a potential risk factor for CVT, current evidence is limited. Larger-scale studies are needed to further clarify this association and inform future clinical recommendations.

## Introduction

Cerebral venous thrombosis (CVT) is an uncommon but potentially devastating form of stroke, accounting for a small proportion of all cerebrovascular events. The pathogenesis of CVT is multifactorial, with both acquired and hereditary prothrombotic conditions contributing to its development. Among the various thrombophilic risk factors, hyperhomocysteinemia has emerged as a potential contributor to venous thromboembolism (VTE) in general, with meta-analyses demonstrating a significant association between elevated plasma homocysteine levels and an increased risk of VTE, including deep vein thrombosis and pulmonary embolism^[^1^]^. However, the specific relationship between hyperhomocysteinemia and CVT remains less clearly defined.

HIGHLIGHTS

- First meta-analysis on hyperhomocysteinemia as a risk factor for cerebral venous thrombosis (CVT).
- Hyperhomocysteinemia increased CVT risk fourfold (OR = 4.17, 95% CI = 2.34–7.43).
- CVT patients had significantly higher homocysteine levels and prevalence (37% vs 15%).
- Findings support screening and correction of homocysteine for CVT risk stratification and prevention.

Several case–control studies have investigated the prevalence of hyperhomocysteinemia in patients with CVT, often reporting higher odds of CVT among individuals with elevated homocysteine levels compared to healthy controls, with odds ratios (ORs) ranging from approximately 4 to 4.6^[^2^]^. These findings suggest a possible link between hyperhomocysteinemia and CVT, although the strength and consistency of this association have not been fully established, as few studies have provided conflicting results^[^3^]^. Furthermore, systematic reviews and meta-analyses of thrombophilia in CVT have identified increased homocysteine as a significant risk factor; however, the clinical utility of routine thrombophilia testing, including homocysteine measurement, remains debated^[^4^]^. Given the potential implications for risk stratification and secondary prevention, a comprehensive synthesis of the available evidence is warranted.

Thus, this systematic review and meta-analysis aim to critically evaluate and quantify the association between hyperhomocysteinemia and the risk of CVT, integrating data from observational studies to inform clinical practice and future research directions.

## Methods

### Study design and registration

The present systematic review was conducted in accordance with the guidelines of the Preferred Reporting Items for Systematic Reviews and Meta-Analysis (PRISMA) 2020 updated statement^[^5^]^. Institutional Review Board approval or informed patient consent was waived for this review. The review protocol was registered in the PROSPERO database. This review aligns with the AMSTAR (Assessing the methodological quality of systematic reviews) guidelines^[^6^]^ and the TITAN guidelines concerning the use of artificial intelligence^[^7^]^.

### Search strategy and selection criteria

A literature search was conducted in English across the PubMed, Embase, Google Scholar, and Scopus databases up to August 2025. The search string was built as follows and was modified according to the database used: (“Hyperhomocysteinemia”[Mesh] OR hyperhomocysteinemia OR homocysteine) AND (“Thrombosis, Intracranial”[Mesh] OR “cerebral venous thrombosis” OR “cerebral vein thrombosis” OR “CVT” OR “sinus thrombosis” OR “venous sinus thrombosis”). The reference list of all selected articles was screened independently to identify additional studies that may have been missed in the initial search. If the publications did not contain all the necessary information, missing information was requested directly from the authors. Care was taken to avoid data duplication. In the initial screening phase, all studies were evaluated for eligibility based on a review of their titles and abstracts. In the second screening phase, full-text articles were reviewed for eligibility criteria. If a study was reported in more than one publication, only one report was included in the meta-analysis. Discussion and consultation were done to settle any disagreements.

**Inclusion criteria**

1. Study with a case–control design
2. Study with confirmed diagnosis of CVT and matched healthy controls (no history of CVT)
3. Study investigating the association between hyperhomocysteinemia and the risk of developing CVT

**Exclusion criteria**

1. Reviews, case reports, abstracts, non-English studies, letters to the editor, and animal studies
2. Absence of a healthy control group
3. Studies lacking sufficient data for extraction or analysis

**Outcome of interest**

1. Comparison of fasting serum homocysteine levels among the CVT patients and the matched healthy controls
2. OR of the risk of developing CVT in patients with hyperhomocysteinemia
3. Prevalence of hyperhomocysteinemia in the CVT patients and the matched healthy controls

### Data extraction

Three investigators reviewed all articles independently. The investigators consulted with one another in cases of differing opinions, and if agreement could not be reached, they consulted other experts. The data extracted from each study included the following: first author, publication year, country, study type, number of cases and controls, mean age, and outcomes of interest. Data cross-checking was performed before analysis.

### Quality assessment

The quality of the included studies was assessed by the Newcastle–Ottawa Scale (NOS)^[^8^]^.The NOS is a star-based system that allows a semi-quantitative assessment of methodological quality in non-randomized studies and consists of eight items grouped into three domains: selection, comparability, and exposure (for case–control studies) or outcome (for cohort studies). The total score ranges from zero to nine stars, with higher scores indicating better methodological quality. Overall, the included studies demonstrated good quality, with consistently strong performance in the selection and comparability domains. However, limitations were commonly observed in the exposure domain, particularly regarding exposure ascertainment, representing the primary source of potential bias across studies.

### Statistical analysis

A meta-analysis was performed using RevMan version 5.4. We conducted a meta-analysis to investigate hyperhomocysteinemia as a risk factor among patients with CVT. Between studies, heterogeneity was assessed using the Chi-squared (χ^2^) test, *I*^2^ statistic, and *I*^2^ greater than 50% with *P* < 0.05 was considered statistically significant. A random or fixed-effects meta-analysis was conducted based on the degree of heterogeneity to pool the OR, prevalence of hyperhomocysteinemia, and the mean difference (MD) in homocysteine level in CVT patients versus healthy controls, along with a 95% confidence interval (CI) from the individual studies. A forest plot was generated to depict the results. A proportional meta-analysis was conducted to determine the prevalence of hyperhomocysteinemia in CVT patients and healthy controls. OR was calculated from the dichotomous data, and MD was calculated for the continuous data. A *P*-value of less than 0.05 was considered statistically significant. Publication bias was assessed using the funnel plot. Sensitivity analysis was conducted to find out the cause of significant heterogeneity.

## Results

### Studies and search results

**Figure 1.**
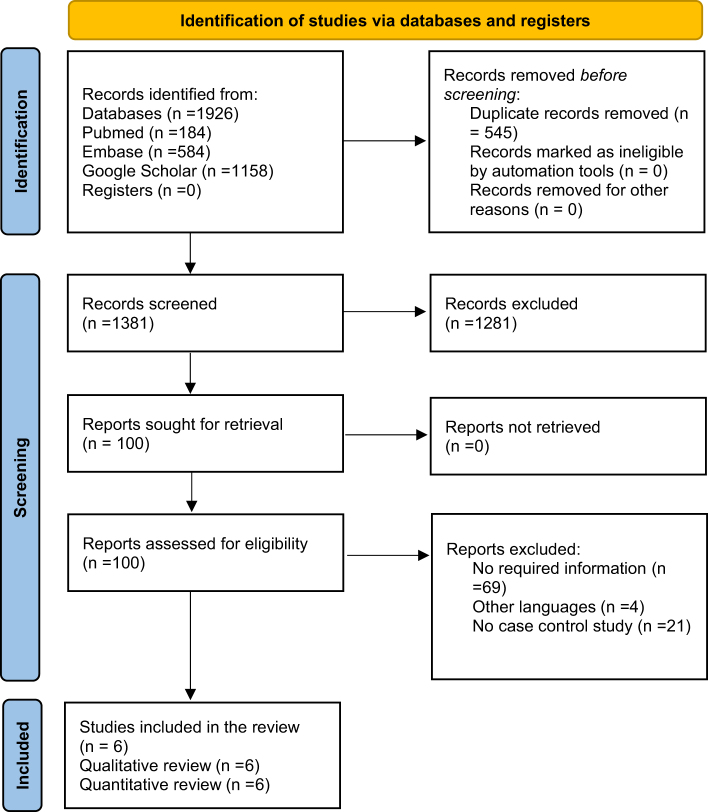
PRISMA flowchart showing the strategy for study selection.

A total of 1926 studies were obtained from the search databases for screening, and duplicate entries in the bibliographic databases were removed. Two authors assessed the remaining studies based on their titles and abstracts. Then, each author conducted a full-text review of a chosen set of studies based on specific inclusion and exclusion criteria. Six individuals were selected for subsequent evaluation to assess their suitability for inclusion. The risk factors for both groups were examined. The common risk factors included puerperium, oral contraceptive use, cigarette smoking, alcohol use, thrombophilia, surgery, and infections. The strategy for study selection has been outlined in Figure 1.

### Study characteristics and the baseline characteristics of the patients

A total of six case–control studies were evaluated, three conducted in Italy and the remaining three in India, Iran, and Mexico^[^3,9–13^]^. These studies involved 1355 participants (455 CVT cases and 900 controls). There were 123 men in the CVT group and 295 men in the control group. The mean age of the participants in the CVT groups ranged from 28 to 48 years, while in the control group, the mean age ranged from 27.45 to 42 years. The detailed study characteristics are outlined in Table 1.

**Table 1:** Detailed study characteristics.

Author, year	Study country	Sample size		Male/Female (%)		Age (years)		Hyperhomocysteinemia definition	Risk factor for CVT		
		CVT	Control	CVT	Control	CVT	Control		Risk factor for CVT	CVT	Control
Bharatkumar *et al*, 2012	India	185	248	32.4/67.4	40.7/59.3	28.91 ± 10.91	27.45 ± 8.27	Hyperhomocysteinemia was defined as the levels of plasma total homocysteine above the 90th percentile of the total homocysteine value distribution in healthy controls.	Puerperium, *n* (%)	80 (43.2)	67 (27.01)
									Cigarette smoking, *n* (%)	24 (12.97)	14 (5.65)
									Alcohol use, n (%)	26 (14.05)	16 (6.45)
									Oral contraceptive use, *n* (%)	11 (5.95)	3 (1.21)
									Family history of vascular disease, *n* (%)	43 (23.24)	17 (6.85)
									Vegetarian diet, *n* (%)	66 (35.7)	91 (36.7)
Cantu *et al*, 2004	Mexico	45	90	15.6/84.4	25.6/74.4	28.0 (14–55) Median(range)	28.0 (16–53) Median(range)	Hyperhomocysteinemia was defined as levels of fasting homocysteine and methionine postload above the 90th percentile of the homocysteine value distribution in the control population.	Current oral contraceptive use, *n* (%)	2 of 37 (5.4)	4 of 66 (6.1)
Martinelli *et al*, 2003	Italy	121	242	24.7/75.3	24.7/75.3	33 (12−64) Median(range)	36 (13−62) Median(range)	Hyperhomocysteinemia was diagnosed when plasma levels of fasting homocysteine or their postmethionine load increments above fasting levels exceeded the 95th percentile of the values obtained in the control group	Smoking, *n* (%)	22 (18)	60 (25)
Taheraghdam *et al*, 2016	Iran	22	36	16.7/83.3	33.3/63.2	32.2 ± 10.8	35.3 ± 11.9	Hyperhomocysteinemia was defined as >90th percentile of homocysteine level of the control group.	NA		
Tufano *et al*, 2014	Italy	56	184	26.7/73.3	27.1/72.9	34.98 ± 11.02	35.05 ± 11.33	Hyperhomocysteinaemia was defined by fasting total homocysteine levels exceeding the 95th percentile of the sex-specific distribution (15.0 μmol/L for men and 12 μmol/L for women) in a control population of healthy subjects from the same geographic area	Prothrombin G201210A	16/55 (29.1%)	10/183 (5.5%)
									Factor V leiden	3/56 (5.4)	15/183 (8.2%)
									Antiphospholipid antibodies	2/47 (4.3%)	5/113 (4.4%)
									Infectious diseases	7/55 (12.7%)	2/183 (1.1%)
									Cancer and hematological disorders	7/54 (13%)	4/183 (2.2%)
									Oral contraceptive users	22/41 (53.7%)	21/132 (15.9%)
									Autoimmune disease	5/55 (9.1%)	7/182 (3.8%)
									Arterial hypertension	9/56 (16.1%)	27/182 (14.8%)
									Hypercholesterolaemia	23/49 (46.9%)	46/159 (28.9%)
									Obesity (BMI ≥30 kg/m^2^)	19/40 (47.5%)	56/144 (38.9%)
									Cigarette smoking	26/55 (47.3%)	67/183 (36.6%)
Boncoraglio *et al*, 2004	Italy	26	100	26.9/73.01	49/51	Mean age 43 years; range 21–73 years)	Men: Mean age 41 years; median 40 years; range 21–67 years) Women: (mean age 42 years; median age 42 years; range 26–72 years)	NA		Oral contraceptive use, Cancer, Carotid surgery	NA

CVT, cerebral venous thrombosis; BMI, body mass index; NA, not available.

### Definition of hyperhomocysteinemia

The definition of hyperhomocysteinemia varied slightly among studies. Bharatkumar *et al* defined it as the levels of plasma total homocysteine above the 90th percentile of the total homocysteine value distribution in healthy controls^[^10^]^. Cantu *et al* defined its levels of fasting homocysteine and methionine postload as above the 90th percentile of the homocysteine value distribution in the control^[^12^]^. Martinelli *et al* defined hyperhomocysteinemia as diagnosed when plasma levels of fasting homocysteine or their postmethionine load (PML) increments above fasting levels exceeded the 95th percentile of the values obtained in the control group^[^13^]^. Taheraghdam *et al* defined it as greater than the 90th percentile or higher of the homocysteine level of the control group^[^11^]^. Tufano *et al* defined hyperhomocysteinemia as fasting total homocysteine levels exceeding the 95th percentile of the sex-specific distribution (15.0 μmol/L for men and 12 μmol/L for women) in a control population of healthy subjects from the same geographic area^[^3^]^. The definition of hyperhomocysteinemia was not found in the study by *Boncoraglio et al*^[^9^]^.

These differences in thresholds and measurement approaches highlight substantial variability in exposure definition across studies, which may have contributed to between-study heterogeneity observed in the pooled analyses.

### Quality assessment results

All six included studies received a score of 7 out of 9 on the NOS, indicating a high-quality study with a low risk of bias (Table 2).

**Table 2:** Risk of bias assessment for case–control studies using the Newcastle–Ottawa Scale.

	Item and score							
Study	Is the case definition adequate?(1)	Representativeness of the cases(1)	Selection of controls(1)	Definition of controls(1)	Comparability of cases and controls on the basis of the design or analysis(2)	Ascertainment of exposure(1)	Same method of ascertainment for cases and controls(1)	Non-Response rate(1)
Bharatkumar *et al*, 2012	1	1	1	1	2	0	0	1
Cantu *et al*, 2004	1	1	1	1	2	0	0	1
Martinelli *et al*, 2003	1	1	1	1	2	0	0	1
Taheraghdam *et al*, 2016	1	1	1	1	2	0	0	1
Tufano *et al*, 2014	1	1	1	1	2	0	0	1
Boncoraglio *et al*, 2004	1	1	1	1	2	0	0	1

**Outcome of interest:** Table 3 describes the outcome of interest in detail.

**Figure 2.**
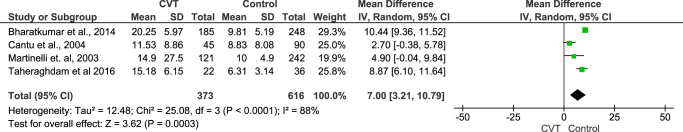
Forest plot showing the homocysteine level among the CVT and healthy control groups.

**Figure 3.**
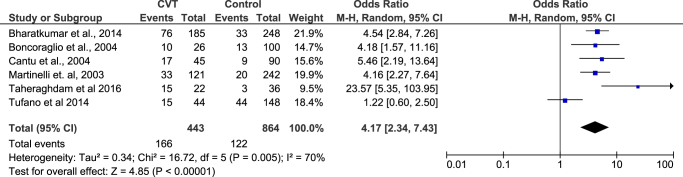
Forest plot showing the risk of CVT with hyperhomocysteinemia.

1. **Comparison of fasting serum homocysteine levels among CVT patients and matched healthy controls**: Four studies compared the serum homocysteine levels among CVT patients and matched healthy controls. The meta-analysis revealed that the serum homocysteine level was higher in the CVT patients as compared with the healthy controls (MD = 7.00 µmol/L, 95% CI = 3.21–10.79 µmol/L) (Fig. 2). The analysis was statistically significant (*P*-value = 0.0003). However, the analysis revealed significant heterogeneity among the included studies (*I*^2^ = 88%, *P*-value < 0.0001). The heterogeneity was reduced to 63% after removing the study by Cantu *et al*, and the analysis remained statistically significant.
2. **OR of risk of developing CVT in patients with hyperhomocysteinemia**: Six studies provided data for calculating the OR of risk of developing CVT in patients with hyperhomocysteinemia. In the 443 CVT cases, there were 166 cases of hyperhomocysteinemia. Similarly, in the 864 controls, there were 122 cases of hyperhomocysteinemia. The meta-analysis of data found the increased (4 times) odds of CVT in hyperhomocysteinemia (OR = 4.17, 95% CI = 2.34–7.43, *P*-value < 0.00001) (Fig. 3). The analysis, although statistically significant, exhibited some heterogeneity (*I*^2^ = 70%). Upon sensitivity analysis, the heterogeneity dropped to 17% after removing Tufano *et al* from the analysis, while maintaining statistical significance.

**Table 3:** Details of homocysteine level and CVT among the CVT patients and the healthy controls.

Study	Fasting homocysteine level (µmol/L)			Hyperhomocysteinemia, *n* (%)			
	CVT	Control	*P*-value	CVT	Control	*P*-value	Adjusted odds of CVT risk
Bharatkumar *et al*, 2012	20.25 ± 25.97	9.81 ± 5.19	<0.001	76 (41.08)	33 (13.31%)	<.001	4.54 (2.74–7.53)
Cantu *et al*, 2004	9.0 (3.04–42.1)^a^	7.0 (2.3–28.7)^a^	0.01	17 (37.8)	9 (10.0)	<0.001	4.6 (1.6–12.8)
Martinelli *et al*, 2003	14.9 ± 27.5	10.0 ± 4.9	.06	33 (27.3)	20 (8.3)	NA	4.2 (2.3–7.6)
Taheraghdam *et al*, 2016	15 (3.9–27.4)^a^	5.95 (1–14.3)^a^	0.001	15 (68.2)	3 (7.9)	0.002	14.3 (2.6–77.1)
Tufano *et al*, 2014	NA	NA	NA	15 (34.1)	44 (29.7)	NA	1.22 (0.59–2.50)
Boncoraglio *et al*, 2004	NA	NA	NA	10 (38.5)	13 (13)	NA	4.18 (1.58–11.16)

NA, not available; CVT, cerebral venous thrombosis.

^a^ Data are in median (range).

**Figure 4.**
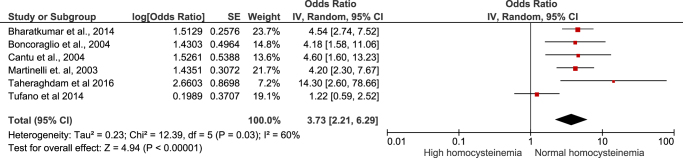
Forest plot showing the pooled odds of CVT with hyperhomocysteinemia.

Next, we pooled the adjusted ORs for the risk of CVT due to hyperhomocysteinemia from the individual studies. We used the generic inverse variance method to pool the ORs. The meta-analysis yielded a pooled adjusted OR of 3.73 (95% CI = 2.21–6.29; *I*^2^ = 60%, *P* < 0.00001) (Fig. 4). Again, removing Tufano *et al* from the analysis, the heterogeneity dropped to 0%, maintaining the statistical significance of the results.

**Figure 5.**
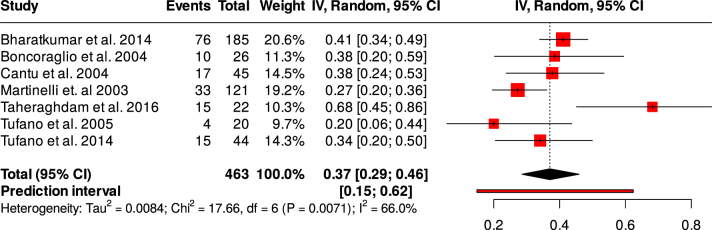
Forest plot showing the prevalence of hyperhomocysteinemia in the CVT patients.

**Figure 6.**
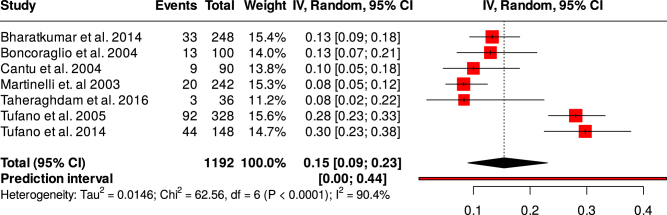
Forest plot showing the prevalence of hyperhomocysteinemia in the healthy controls.

3. **Prevalence of hyperhomocysteinemia in CVT patients and matched healthy controls**: Next, we performed a proportional meta-analysis to determine the prevalence of hyperhomocysteinemia in CVT patients and matched healthy controls. The analysis revealed a proportion of hyperhomocysteinemia in the CVT patients of 37% (95% CI = 29–46%, *I*^2^ = 66%) (Fig. 5). However, the prevalence of hyperhomocysteinemia in the healthy controls was relatively lower (15%, 95% CI = 9–23%, *I*^2^ = 90.4%) (Fig. 6).
4. **Qualitative analysis**: Folate and vitamin B12 are interrelated with the metabolism of homocysteine. Accordingly, Cantu *et al* estimated the risk of CVT associated with low folate and low Vitamin B12 levels, and the risks found were statistically significant (OR = 3.5, 95% CI = 1.2–10.0) and OR = 5.1, 95% CI = 1.8–14.2, respectively. In the same study, both the folate level and vitamin B12 level were significantly lower in the CVT group as compared with the control group. Low vitamin B12 levels were found to be a risk factor for CVT in the study by Taheraghdam *et al* but not low folate levels (OR = 24.6, 95% CI = 2.3–262.9) and (OR = 3.5, 95% CI = 0.4–31.9), respectively. In the same study, vitamin B12 levels were significantly lower in the CVT patients compared with the control; however, the folate level did not differ. In contrast, Martinelli *et al* found no association between low folate and low vitamin B12 and the risk of CVT. Accordingly, the folate and vitamin B12 levels did not differ between the two groups.

## Discussion

This meta-analysis demonstrates a significant association between hyperhomocysteinemia and CVT, supporting the role of hyperhomocysteinemia as an important biochemical risk marker^[^2,9,12,13^]^. Meta-analyses of venous thrombosis in general indicate that individuals with the disease have approximately a 2.5-fold higher odds of elevated fasting homocysteine and a 2.6-fold higher odds of an abnormal post-methionine homocysteine response compared with controls, indicating a significant association between hyperhomocysteinemia and venous thrombosis^[^1^]^. While elevated homocysteine is a recognized risk factor for several vascular thromboses, hyperhomocysteinemia may exert a more pronounced pathogenic effect on cerebral venous circulation than on other vascular territories (ischemic stroke, pulmonary embolism, deep vein thrombosis, etc.)^[^1,2,14–17^]^. The American Heart Association/American Stroke Association (AHA/ASA) guidelines also summarizes these findings, noting that hyperhomocysteinemia is a significant risk factor for CVT, with ORs consistently above 4 in case–control studies^[^2^]^.

The association between hyperhomocysteinemia and CVT demonstrates notable variation by ethnicity, geographic location, nutrition, sex, and age. Their association across different populations has a prevalence rate ranging from 27% to 52%^[^9,13,18^]^. Studies from India and Mexico report a high prevalence, with ORs of 4.2–4.6, and a substantial proportion of cases attributable to vitamin B12 deficiency and the MTHFR C677T polymorphism, particularly in populations with dietary patterns that predispose to low B12 levels (e.g., vegetarianism)^[^18,19^]^. In contrast, data from Iran indicate that while elevated homocysteine is a significant risk factor for CVT, the MTHFR 677TT genotype itself is not directly associated with CVT risk, but rather serves as a determinant of higher homocysteine levels^[^20^]^. European studies (e.g., Italy) also report a strong association between hyperhomocysteinemia and CVT but do not consistently find a link with the MTHFR genotype or low folate levels independent of homocysteine levels^[^2^]^. These findings suggest that the impact of hyperhomocysteinemia on CVT risk is modulated by local genetic and nutritional factors (folate, vitamin B12, and vitamin B6), with higher attributable risk in regions where vitamin deficiencies and MTHFR polymorphisms are prevalent. These vitamins are essential cofactors in homocysteine metabolism: folate and vitamin B12 are required for remethylation of homocysteine to methionine, while vitamin B6 is necessary for transsulfuration to cysteine^[^2,18,21,22^]^. Hyperhomocysteinemia is a significant risk factor for CVT in both sexes, but some studies report a higher frequency in men compared to women (59% vs 41%)^[^18^]^. In women, pregnancy and the puerperium are established prothrombotic states, and hyperhomocysteinemia further amplifies CVT risk in this context. Case–control data from India demonstrate that hyperhomocysteinemia confers a markedly increased risk for puerperal CVT (adjusted OR 10.8), independent of folate and B12 levels^[^19^]^. However, isolated female-specific risk factors (FSRFs) such as pregnancy or oral contraceptive use are rarely the sole contributors; most affected women have additional prothrombotic risk factors^[^18^]^. The clinical severity and outcomes of CVT do not differ significantly between men and women, nor between women with and without FSRFs^[^18^]^. The medical literature does not provide direct comparative data on the prevalence of hyperhomocysteinemia in younger (<40 years) versus older CVT patients. However, studies from India and Iran include broad age ranges (e.g., 20–63 years) and report high rates of hyperhomocysteinemia across all adult age-groups^[^18,20^]^. Given that CVT itself is more common in younger adults and that nutritional deficiencies and genetic predispositions may be more relevant in younger populations, it is plausible that hyperhomocysteinemia is at least as prevalent, if not more so, in younger patients with CVT; however, this is not explicitly confirmed in the cited literature.

An important source of heterogeneity in the present meta-analysis is the variability in the definition of hyperhomocysteinemia across included studies. Definitions ranged from percentile-based thresholds (90th vs 95th percentile), fasting-only measurements, combined fasting and PML increments, sex-specific cutoffs, and, in one study, the absence of a clearly reported definition. Such heterogeneity in exposure classification may reduce comparability across studies and introduce non-differential misclassification bias. This type of misclassification would be expected to bias effect estimates toward the null; therefore, the observed strong association between hyperhomocysteinemia and CVT may represent a conservative estimate of the true effect. Additionally, differing thresholds and measurement protocols likely contributed to the substantial between-study heterogeneity observed in the pooled ORs and MDs, despite the use of random-effects models and sensitivity analyses.

Another important consideration is the substantial heterogeneity observed in several pooled analyses, with *I*^2^ values ranging from 66% to 90%. Sensitivity analyses identified specific studies – most notably Cantu *et al* and Tufano *et al* – as primary contributors to this variability. Differences in study populations, geographic and nutritional factors, and the methods used to define hyperhomocysteinemia likely account for the observed heterogeneity. For instance, Tufano *et al* used sex-specific thresholds, whereas Cantu *et al* combined fasting and PML measurements, which may have led to systematic differences in classification. These methodological differences suggest that while the pooled effect estimates indicate a strong association between hyperhomocysteinemia and CVT, caution is warranted when interpreting the magnitude of the effect.

The pathophysiology of elevated homocysteine levels and venous thrombosis operates through several interrelated mechanisms involving endothelial dysfunction, platelet activation, and alterations in the coagulation cascade. Homocysteine induces oxidative stress, leading to increased lipid peroxidation and direct endothelial injury, which impairs the antithrombotic properties of the vascular endothelium and promotes a procoagulant state^[^21,22^]^. This endothelial damage facilitates the expression of adhesion molecules and tissue factor, enhancing leukocyte and platelet adhesion and activation. Hyperhomocysteinemia also increases platelet activation, as evidenced by elevated urinary markers such as 11-dehydro-thromboxane B2, and this effect is at least partially reversible with folic acid supplementation^[^21,22^]^. Additionally, homocysteine interferes with the balance of coagulation and fibrinolysis by increasing factor V activity, reducing protein C activation, and impairing antithrombin III function, thereby favoring thrombosis^[^21^]^. MTHFR gene polymorphisms, particularly the C677T variant, are key genetic determinants of elevated homocysteine levels. The C677T variant reduces MTHFR enzyme activity, leading to impaired remethylation of homocysteine to methionine and subsequent accumulation of homocysteine, especially in the context of low folate status^[^18,20–23^]^. Individuals homozygous for the 677TT genotype have significantly higher plasma homocysteine concentrations compared to heterozygotes or wild-type individuals, and this genotype is associated with increased oxidative stress and platelet activation^[^18,20,22^]^. The A1298C polymorphism has a less pronounced effect on homocysteine levels and is not consistently associated with an increased risk of VTE^[^23^]^. While the C677T polymorphism is a determinant of hyperhomocysteinemia, its direct association with CVT risk is less clear; some studies and meta-analyses suggest an increased risk in Asian populations but not consistently in Caucasian cohorts^[^2,20,23^]^.

The AHA/ASA guidelines for CVT note that hyperhomocysteinemia is associated with an increased risk of CVT, but they do not recommend routine homocysteine testing in CVT patients^[^2^]^. Similarly, the 2021 AHA/ASA guidelines for secondary stroke prevention acknowledge a modest reduction in stroke risk with B-vitamin supplementation in certain populations but does not specifically endorse homocysteine testing or treatment for CVT or other venous thrombotic events^[^24^]^. Recent data also suggest that *S*-adenosylhomocysteine (SAH), a metabolic precursor of homocysteine, may confer an even higher risk for CVT, with an OR of 35.77 (95% CI = 19.45–65.79), although the clinical utility of SAH measurement requires further validation^[^25^]^.

Folic acid supplementation has been shown to reduce homocysteine levels and reverse associated oxidative stress and platelet activation, supporting its role in the primary prevention of thrombotic events in susceptible individuals^[^2,18,21,22^]^. The evidence for lowering homocysteine levels with folate and B-vitamin supplementation to reduce the risk of CVT is limited and inconclusive. While meta-analyses and Mendelian randomization studies suggest that B-vitamin supplementation can modestly reduce stroke risk, particularly in populations without folate fortification and with longer follow-up, these findings are primarily relevant to arterial stroke rather than CVT^[^26–28^]^. Observational studies demonstrate a strong association between hyperhomocysteinemia and CVT risk, and B-vitamin deficiencies are common contributors, but randomized trials have not established that homocysteine-lowering therapy reduces the risk of first or recurrent venous thrombosis, including CVT^[^13,15,17^]^. The medical literature emphasizes that while severe hyperhomocysteinemia due to inherited disorders or profound vitamin deficiency should be identified and treated, routine screening and supplementation for moderate elevations in homocysteine have not been shown to reduce venous thrombotic risk in randomized trials^[^15,17^]^.

## Conclusion

To determine if homocysteine-lowering therapy can prevent the recurrence of CVT, future research must transition from observational studies to well-designed randomized controlled trials (RCTs). While existing data show a link between hyperhomocysteinemia and thrombosis, RCTs are required to rule out confounding factors and establish a definitive causal benefit^[^15,17,29,30^]^. A precision medicine approach is recommended, integrating genetic analysis of MTHFR and CBS mutations with biochemical assessments to account for interindividual variability in metabolism. By stratifying patients based on these genetic and metabolic profiles, researchers can better identify specific subgroups likely to benefit from targeted B-vitamin interventions, ultimately leading to more effective secondary prevention strategies^[^17,21,29,31^]^.

### Strengths and limitations

The strength of the study is that it is the first meta-analysis on the topic. Also, this study uses multiple databases. Our pooled estimates provide a more comprehensive assessment by integrating data from multiple studies, clarifying the strength and consistency of the association while accounting for variability across individual studies. A few limitations of this study should be considered. The included studies are of a limited number. First, the sample size of the included patients was considerably low. Some of the analyses revealed considerable heterogeneity. Furthermore, the definitions of hyperhomocysteinemia varied considerably among the included studies, encompassing different percentile thresholds, measurement protocols, and sex-specific cutoffs. This heterogeneity in exposure definition may have contributed to between-study variability and introduced non-differential misclassification bias, warranting cautious interpretation of the pooled estimates.

## Data Availability

The data generated in this study are available upon request from the corresponding author.

## Research registration unique identifying number (UIN)

PROSPERO Registration ID: CRD420251145355.

## Peer and provenance statement

Not commissioned, externally peer-reviewed.
